# Prognostic Value of Elevated miR‐502‐3p in Patients With Post‐Stroke Cognitive Impairment

**DOI:** 10.1002/brb3.71208

**Published:** 2026-02-09

**Authors:** Yi Lin, Liang Xu, Yuhao Zhang, Cui Zou

**Affiliations:** ^1^ Department of Nursing The Yancheng Clinical College of Xuzhou Medical University, The First People's Hospital of Yancheng Yancheng China; ^2^ Neuroscience Medical Center, Chongqing No. 13 People's Hospital Chongqing China; ^3^ Department of Rehabilitation Medicine Nanjing Lishui District Hospital of Traditional Chinese Medicine Nanjing China; ^4^ Department of Rehabilitation Medicine, Changde Hospital, Xiangya School of Medicine Central South University (The First People's Hospital of Changde City) Changde China

**Keywords:** miR‐502‐3p, post‐stroke cognitive impairment, prognosis

## Abstract

**Background:**

Post‐stroke cognitive impairment (PSCI) is a common complication following a stroke. Recent findings highlight the role of miR‐502‐3p in both vascular and neurodegenerative diseases. However, the role in PSCI remains uncovered.

**Objective:**

This study emphasized the differential expression of miR‐502‐3p and subsequently evaluated the predictive value of miR‐502‐3p expression levels for PSCI.

**Materials and methods:**

The study subjects included 112 patients with PSCI and 161 individuals with post‐stroke cognitive normality. The relative expression of miR‐502‐3p was calculated by qPCR, while its predictive value for PSCI was assessed via ROC curve. Pearson's correlation coefficient was utilized to analyze the correlation between serum miR‐502p‐3p levels and PSCI. Multivariate logistic regression was used to identify risk factors of PSCI.

**Results:**

Serum miR‐502‐3p was identified as significantly elevated in the PSCI group. The area under the ROC curve was 0.850, with a sensitivity of 76.79% and a specificity of 77.64%. The miR‐502‐3p level was positively correlated with both NIHSS and mRS scores. In addition, a negative correlation was observed between the miR‐502‐3p level and MoCA score. Elevated miR‐502‐3p and hypertension were identified as independent risk factors for PSCI.

**Conclusion:**

Significantly elevated serum miR‐502‐3p was a promising biomarker for the onset of PSCI. Elevated miR‐502‐3p and hypertension were independent risk factors for PSCI.

## Graphical abstract

Significantly elevated serum miR‐502‐3p was positively correlated with both NIHSS and mRS scores, while it was negatively related to the MoCA score. Furthermore, the serum miR‐502‐3p was identified as a promising biomarker post‐stroke cognitive impairment (PSCI). Multivariate logistic regression confirmed it as an independent risk factor for PSCI.

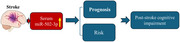

## 1 Introduction

Stroke, commonly known as “brain attack,” results from a sudden cerebrovascular burst or blockage, causing damage to brain tissue and even death (Zhao et al. 2022). It is a major global health concern. The World Stroke Organization (2022 version) has declared that stroke imposes a financial burden equivalent to 0.66% of global GDP, totaling over $721 billion. And from 1990 to 2019, the incidence of stroke increased by 70% and mortality by 43% (Feigin et al. 2022). Therefore, stroke is a major health issue with substantial economic implications, posing considerable challenges to healthcare systems and decisions.

It has been reported that stroke is a highly disabling disease (Farzadfard et al. 2019). Post‐stroke cognitive impairment (PSCI) is a common complication following stroke, which involves a significant decline in executive function and difficulties like memory, attention, and language. Increasing evidence has indicated stroke raises both the risk and severity of cognitive impairment (Y. Y. Huang et al. 2022). Studies have found that nearly a third of stroke patients face cognitive issues afterward (Li et al. 2023). There is also evidence that over half of stroke patients develop cognitive impairments within 6 months, which is linked to increased disability and poorer quality of life (Merriman et al. 2019). Additionally, PSCI can negatively affect a patient's rehabilitation, increasing the risk of disability (Elendu et al. 2023). Thus, the early identification of high‐risk individuals for PSCI is crucial for improving recovery and quality of life and for reducing the burdens in economy and caring.

The miR‐502‐3p is known to be involved in various prevalent human diseases, including cancer, diabetes, and leukemia (Devara et al. 2023; Ruiz‐Lafuente et al. 2015). In a study about Alzheimer's disease, miR‐501‐3p is reported to be predominantly expressed in astrocytes (Devara et al. 2025). Recent findings highlight the role of miR‐502‐3p in vascular diseases. For instance, abnormally elevated miR‐502‐3p has been identified as a biomarker for aortic translocation (Abu‐Halima et al. 2022). Emerging reports on neurodegenerative diseases have shown its regulatory role in cognitive impairment. For example, miR‐502‐3p can effectively identify those with frontotemporal dementia among healthy people (Grasso et al. 2019). Furthermore, a study on Alzheimer's disease highlights miR‐502‐3p's role in cognition by modulating glutamatergic function and synapses (Devara et al. 2023). Currently, miR‐502‐3p was found to be upregulated in patients with PSCI. Together, these findings indicated that this microRNA impacted cognitive abilities by altering glutamatergic function and synaptic properties. Thus, we speculated that miR‐502‐3p might also link to PSCI. In PSCI diagnostic research, miR‐511‐3p and miR‐132 have shown higher diagnostic sensitivity and specificity than miR‐502‐3p (S. Huang et al. 2016; T. Wang et al. 2024). M. Yuan et al. (2022) evaluated miR‐21, miR‐132, and miR‐200b in PSCI, finding that miR‐132/200b had low sensitivity, miR‐21 had high sensitivity/specificity, and the three combined outperformed single‐miRNA detection. Given this evidence, future studies should focus on evaluating multi‐miRNA combined detection schemes, which better meet clinical accuracy/reliability needs and may offer more practical references for PSCI prognosis than single indicators.

This study emphasized the differential expression of miR‐502‐3p and subsequently evaluated the predictive value of miR‐502‐3p expression levels for PSCI from many aspects, by analyzing serum samples and clinical data from stroke patients. The findings could provide a new theoretical basis for predicting the occurrence of PSCI.

## 2 Materials and Methods

### 2.1 Study Object and Serum Preparation

Our study subjects were first‐time ischemic stroke patients from Nanjing Lishui District Hospital of Traditional Chinese Medicine, comprising 112 patients with PSCI and 161 individuals with post‐stroke cognitive normality (PSCN) during the same period. Ethical approval (No. 2023LW005) and informed consent were obtained before the experiment. All subjects met the stroke diagnostic criteria (Panni et al. 2019), and PSCI was diagnosed according to relevant standards (Quinn et al. 2021). The inclusion criteria were (1) first‐time stroke diagnosed by magnetic resonance imaging and clinical evaluation, (2) Montreal cognitive assessment scale (MoCA) score <26, (3) complete clinical data, (4) cognitive impairment appeared after stroke and lasted at least 3 months, and (5) cognitive or communication issues before or during stroke onset. The exclusion criteria were (1) history of stroke, (2) neurological diseases, and (3) other diseases causing cognitive impairment, such as infections as well as tumors. For the PSCN group, MoCA score was >26, with other conditions matching the PSCI group. Basic information was extracted from electronic medical records. National Institutes of Health Stroke Scale (NIHSS) scores were assessed and recorded at admission. Peripheral blood samples, collected after admission within 48 h of acute ischemic stroke (AIS) onset, were left at room temperature for 30 min, then centrifuged at 3500 rpm for 15 min to obtain serum. The samples were carefully labeled and stored at −80°C for later analysis.

### 2.2 Rehabilitation Training

All patients received treatment in accordance with the 2018 Chinese Guidelines for the Diagnosis and Treatment of Acute Ischemic Stroke, including thrombolysis, antiplatelet therapy, circulation improvement, neuroprotection, free radical scavenging, and statin administration. Then, rehabilitation interventions were performed within 2 days of vital sign stabilization. The training program encompassed sitting and lying position exercises; joint range of motion improvement, muscle strength enhancement, and gait training; maintenance of proper limb positioning, turning, transfer exercises, and passive limb movements to optimize joint mobility; as well as cognitive and social adaptability training. Training items and intensity were individualized based on each patient's condition, with the program conducted continuously for 14 days.

### 2.3 Reverse Transcription Quantitative Polymerase Chain Reaction

The miRNAs isolated from serum samples were accomplished by the miRNeasy Serum Kit. Briefly, the lysis buffer was mixed with serum samples and transferred to a silica membrane centrifuge column. After centrifugation and washing, miRNA was eluted with RNase‐free water at an appropriate temperature. The entire process should be free of RNase contamination. A reverse transcription process was necessary to obtain cDNA. A SYBR GREEN‐based qPCR kit was employed to calculate the relative expression of miR‐502‐3p. The Ct values of the target miR‐502‐3p were normalized to the Ct values of U6 RNA before quantification using the 2^−^^ΔΔCt^ method. U6 was determined as most stable reference gene for each sample using standard algorithms GeNorm (Vandesompele et al. 2002). The basic conditions included initial denaturation and subsequent 38 cycles of denaturation, annealing as well as extension.

### 2.4 Cognitive Appraisal

Stroke severity was assessed by NIHSS scores at admission, covering 10 aspects including consciousness, limb sensation as well as coordination. The total score was 42, with higher scores indicating more severe neurological damage. The MoCA, scored 3 months after stroke, evaluated cognitive functions such as language, attention, visuospatial skills, executive function, abstract thinking, calculation, and memory. The total score was 30, and a score below 26 indicated cognitive impairment. The modified Rankin scale (mRS) score focused on daily living abilities and functional recovery, with six levels. A higher score signified more severe functional deficits.

### 2.5 Statistical Analysis

Data, processed by SPSS 21.0 or GraphPad Prism 7.0, was presented as mean ± SD or counts and percentages. Kolmogorov–Smirnov statistics were used to test the normal distribution of continuous variables. After Kolmogorov–Smirnov testing, normally distributed continuous variables (such as age, body mass index [BMI], miR‐502‐3p expression level, MoCA, and NIHSS) were compared with Student's *t*‐test, whereas non‐normally distributed variables (such as mRS score) were analyzed with the Mann–Whitney *U* test. Categorical variables (such as gender, hypertension, and diabetes) were compared by the chi‐square test. The predictive value of miR‐502p‐3p for PSCI was assessed via receiver operator characteristic (ROC) curve. In correlation analyses between miR‐502‐3p levels and PSCI‐related variables, Pearson's correlation coefficient was employed for normally distributed data, while Spearman's rank correlation was applied for non‐normally distributed variables (such as mRS score). Multivariate logistic regression was performed to identify independent risk factors of PSCI. All continuous data are presented as mean ± standard deviation. *p* value <0.05 indicated a significant statistical difference.

## 3 Results

### 3.1 Demographics of Included Participants

As shown in Table 1, comparison by Student's *t*‐test on average age (*p* = 0.116) and BMI, (*p* = 0.659) showed no statistical difference between the groups. Furthermore, the chi‐square test on gender (*p* = 0.432), hyperlipidemia (*p* = 0.886), smoking (*p* = 0.700), drinking (*p* = 0.820), previous stain (*p* = 0.937) or antiplatelet (*p* = 0.675) therapy, site of infarction (*p* = 0.997), and stroke subtype (*p* = 0.928) also proved no statistical difference between the two groups. By contrast, the proportions of diabetes (*p* = 0.029), hypertension (*p* = 0.019), and education level (*p* = 0.016) were significantly higher in the PSCI group than that of the PSCN group.

**TABLE 1:** Basic characteristics of included participants.

Indicators	PSCI (*n* = 112)	PSCN (*n* = 161)	*p* value
Age (years)	58.04 ± 8.05	56.48 ± 8.07	0.116
BMI (kg/m^2^)	22.67±2.37	22.54±2.45	0.659
Gender, *n* (%)			0.432
Male	57 (50.89)	74 (45.96)	
Female	55 (49.11)	87 (54.04)	
Diabetes, *n* (%)	47 (41.96)	47 (29.19)	0.029
Hypertension, *n* (%)	57 (50.89)	60 (37.27)	0.019
Hyperlipidemia, *n* (%)	35 (31.25)	49 (30.43)	0.886
Education level (years)	6.34 ± 3.67	7.60 ± 4.57	0.016
Smoking, *n* (%)	59 (52.68)	81 (50.31)	0.700
Drinking, *n* (%)	60 (53.57)	84 (52.17)	0.820
Previous statin therapy, *n* (%)	17 (15.18)	25 (15.53)	0.937
Previous antiplatelet therapy, *n* (%)	22 (19.64)	35 (21.74)	0.675
Acute infarct volume (mL)	2.29 ± 1.03	2.15 ± 0.91	0.247
Site of infarction, *n* (%)			0.997
Basal ganglia	19 (16.96)	23 (14.29)	
Frontal	19 (16.96)	31 (19.25)	
Insula	15 (13.39)	21 (13.04)	
Occipital	9 (8.04)	13 (8.07)	
Parietal	15 (13.39)	22 (13.66)	
Temporal	23 (20.54)	35 (21.74)	
Thalamus	12 (10.71)	16 (9.93)	
Stroke subtype, *n* (%)			0.928
Atherosclerotic	41 (36.61)	55 (34.16)	
Cardioembolic	18 (16.07)	30 (18.63)	
Small vessel occlusion	39 (34.82)	54 (33.54)	
Others	14 (12.50)	22 (13.67)	

Abbreviations: BMI, body mass index; PSCI, post‐stroke cognitive impairment; PSCN, post‐stroke cognitive normality.

### 3.2 Relative miR‐502‐3p Level and Predictive Efficiency

**FIGURE 1:**
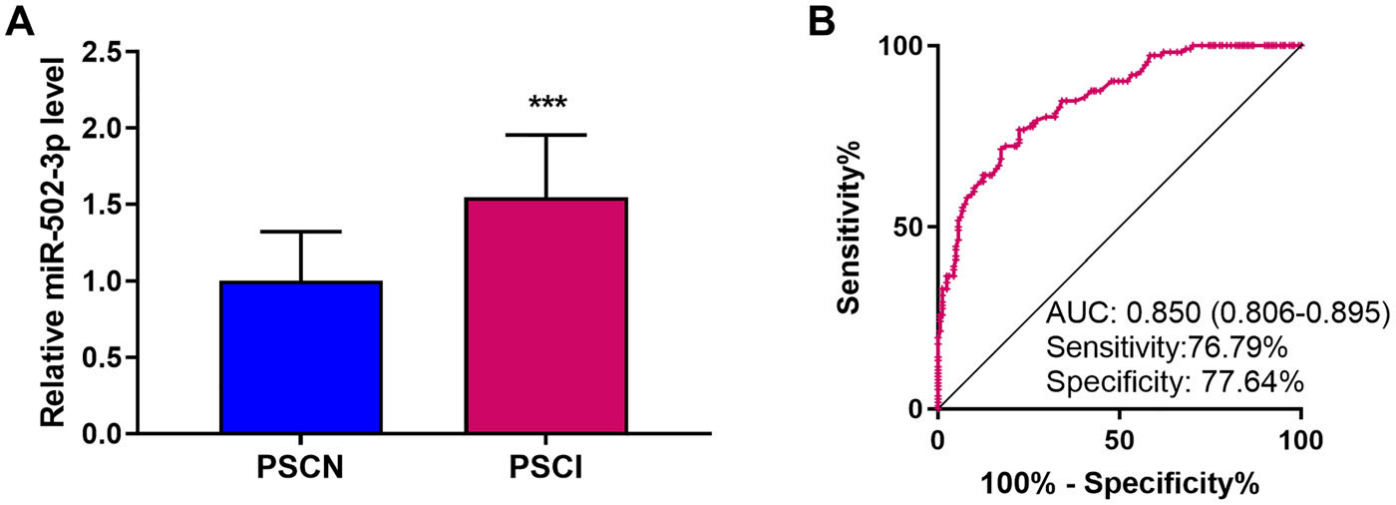
Relative expression of miR‐502‐3p and its predictive value for post‐stroke cognitive impairment (PSCI). (A) Relative expression of miR‐502‐3p. The statistical difference between the two groups was compared using Student's *t*‐test. (B) Receiver operator characteristic curve (ROC) curve ofmiR‐502‐3p. The data were presented as mean ± SD. ***p < 0.001.

Compared to the PSCN group, the relative expression level of serum miR‐502‐3p was significantly elevated in the PSCI group (Student's *t*‐test, Figure 1A, *p* < 0.001). The ROC curve generated based on the relative expression of serum miR‐502‐3p was employed to evaluate its predictive value for PSCI. The results revealed that the area under the ROC curve was 0.850 (95% confidence interval [CI]: 0.806–0.895), with a sensitivity of 76.79% and a specificity of 77.64% (Figure 1B). The positive predictive value and negative predictive value were 69.35% and 82.55%, respectively. These results suggested that elevated serum miR‐502‐3p level was highly predictive of the occurrence of PSCI.

### 3.3 Correlation Analysis of miR‐502‐3p With PSCI

**FIGURE 2:**
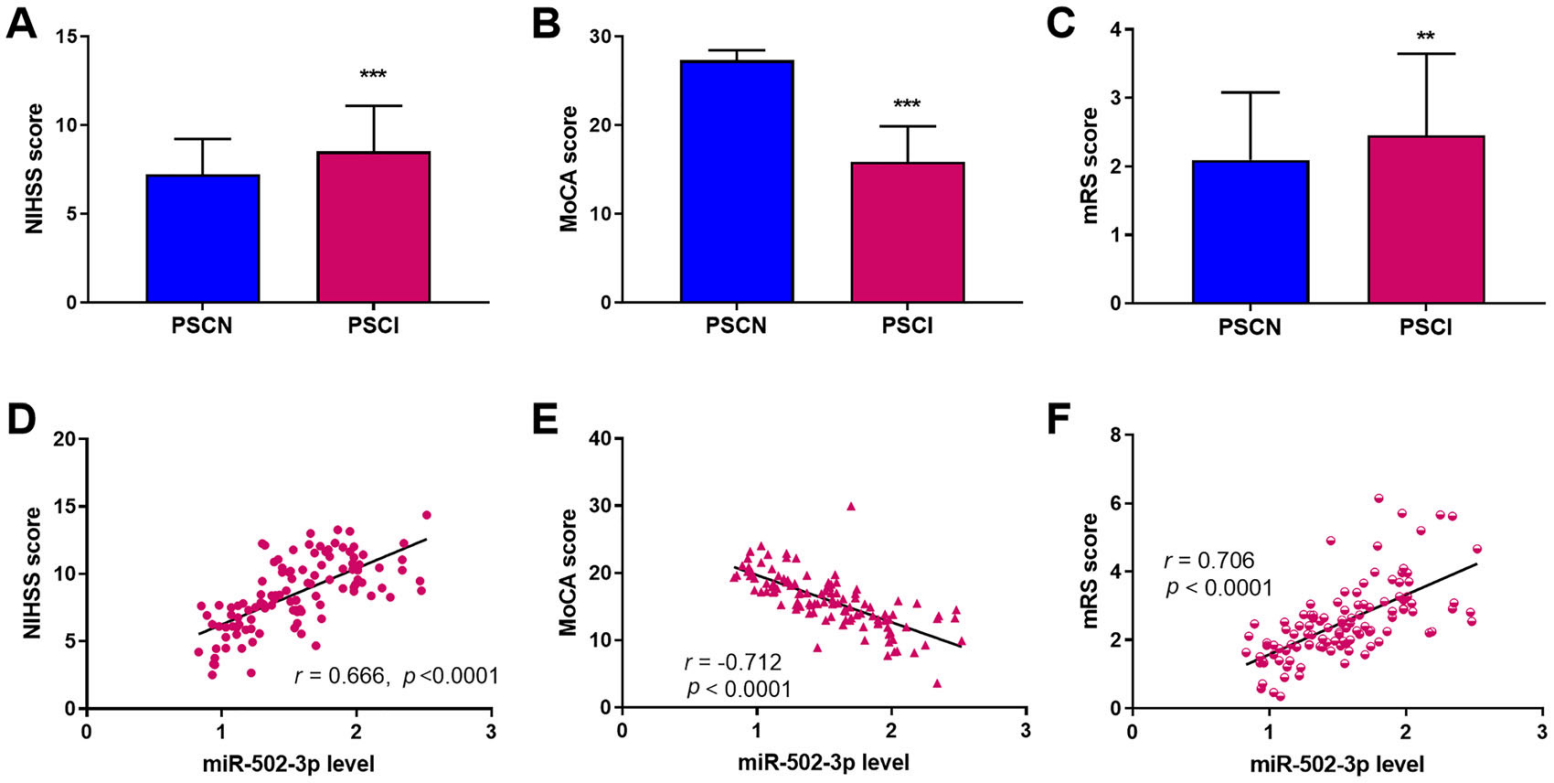
Correlation analysis of miR‐502‐3p with PSCI. (A) National Institutes of Health Stroke Scale of the United States (NIHSS) score in the post‐stroke cognitive normality (PSCN) and PSCI groups. The data were compared using Student's *t*‐test. (B) Montreal Cognitive Assessment Scale (MoCA) score in the PSCN and PSCI groups. The data were compared using Student's *t*‐test. (C) modified Rankin scale (mRS) score in the PSCN and PSCI groups. The Mann–Whitney *U* test was employed for comparison concerning mRS score. (D) Correlation analysis of miR‐502‐3p and NIHSS score. The data were assessed by Pearson's correlation coefficient. (E) Correlation analysis of miR‐502‐3p and MoCA score. The data were assessed by Pearson's correlation coefficient. (F) Correlation analysis of miR‐502‐3p and mRS score. The data were assessed by Spearman's rank correlation. The data were presented as mean ± SD. **p < 0.01, ***p < 0.001.

The NIHSS score at admission for stroke was first compared, and the results manifested a higher average score in the PSCI group (Student's *t*‐test, Figure 2A, *p* < 0.001). Moreover, the average MoCA score was significantly decreased (Student's *t*‐test, Figure 2B, *p* < 0.001), while the average mRS score was increased (Mann–Whitney *U* test, Figure 2C, *p* < 0.01). Correlation analysis revealed that the miR‐502‐3p level positively correlated with both NIHSS (Figure 2D, Pearson *r* = 0.666, *p* < 0.0001) and mRS scores (Figure 2F, Spearman *r* = 0.706, *p* < 0.0001). In addition, a negative correlation was observed between the miR‐502‐3p level and MoCA score (Figure 2E, Pearson *r* = −0.712, *p* < 0.0001). The above data indicated that serum miR‐502‐3p level was closely correlated with the severity of stroke and cognitive impairment.

### 3.4 Risk Factor for PSCI

**FIGURE 3:**
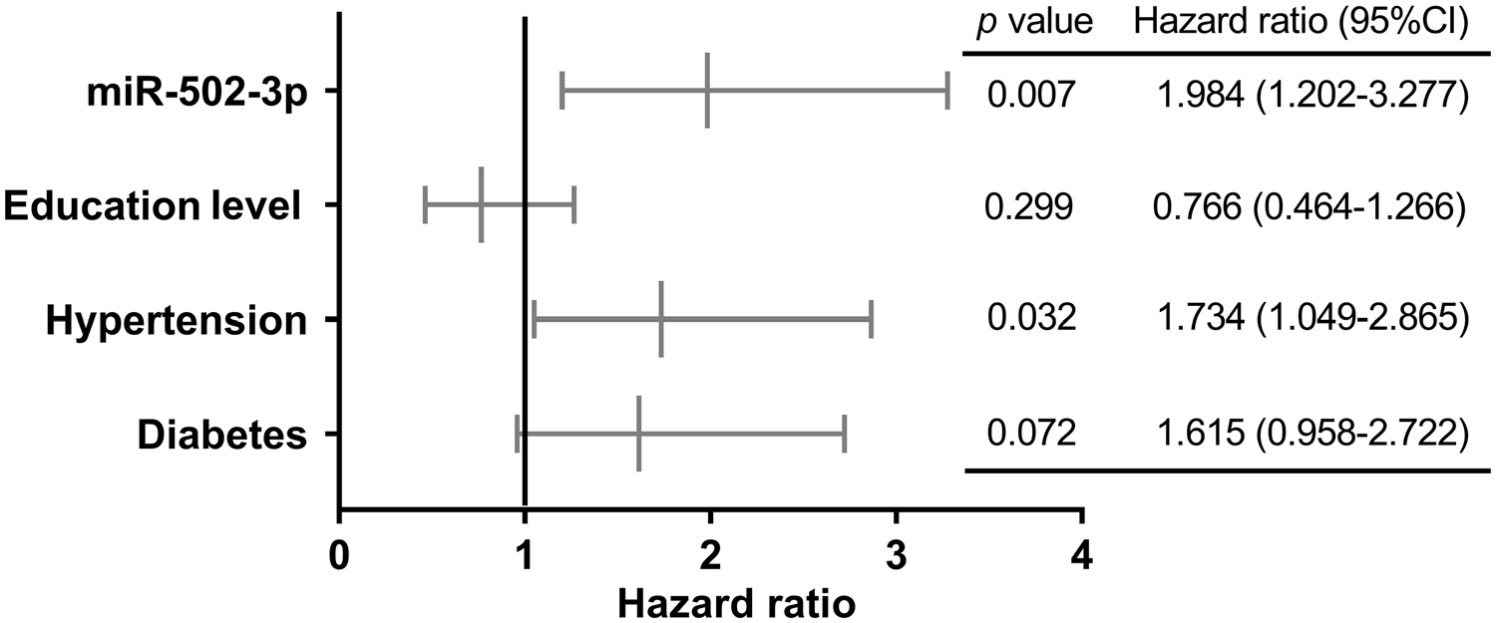
Forest diagram of risk factor for PSCI.

Multivariate logistic regression analysis was employed to screen risk factors of PSCI as shown in Figure 3, and all significant univariate variables were included in the logistic regression model. The model's goodness of fit was evaluated with the Hosmer–Lemeshow test (*p* = 0.921). The results indicated that the PSCI risk was not related to education level (HR [hazard ratio] = 0.766, 95% CI: 0.464–1.266, *p* = 0.299) and diabetes (HR = 1.615, 95% CI: 0.958–2.722, *p* = 0.072). In addition, elevated miR‐502‐3p boosted the risk of PSCI by 98.4%, and hypertension raised it by about 73.4%. Thus, elevated miR‐502‐3p (HR = 1.984, 95% CI: 1.202–3.277, *p* = 0.007) and hypertension (HR = 1.734, 95% CI: 1.049–2.865, *p* = 0.032) were identified as independent risk factors for PSCI.

## 4 Discussion

Currently, PSCI is mainly diagnosed through clinical assessment, neuropsychological tests, and imaging evaluation (Sloane et al. 2023; Xu et al. 2023). However, these methods are applicable within a certain period after stroke and can be easily affected by subjective factors. In comparison, specific molecules in blood or cerebrospinal fluid can predict PSCI risk earlier. For instance, studies have found that miR‐132 levels may change in the early stage of PSCI, thus aiding early diagnosis (Q. Lu et al. 2024). The data from our research indicated that serum miR‐502‐3p was significantly elevated in the PSCI group, and the subsequent ROC curve confirmed the promising predictive value for PSCI. The miR‐502‐3p level was positively correlated with both NIHSS and mRS scores, while it was negatively correlated with the MoCA score. Elevated miR‐502‐3p and hypertension were identified as independent risk factors for PSCI.

Stroke can induce local cerebral ischemia and hypoxia. It not only impairs energy metabolism and causes neuronal damage or death but also triggers a cascade of secondary injuries that culminate in cognitive dysfunction (E and Wang 2024; Tian et al. 2022). Literature has documented that serum miRNA levels show significant dynamic changes in different post‐stroke phases, closely associated with stroke's pathophysiological progression. For example, in patients with diabetes mellitus complicated by AIS or transient ischemic attack (TIA), both miR‐195‐5p and miR‐451a are twofold upregulated in the acute phase compared to non‐diabetic patients, with gradual reduction at 24 and 72 h post‐onset. Notably, in the non‐diabetic TIA subgroup, these two miRNAs are higher than in the control group but tend to fully attenuate after 72 h (Giordano et al. 2020). Our study centered on the early predictive value of miR‐502‐3p in PSCI. Accordingly, blood samples were collected within 48 h of stroke onset. In this study, we found that serum miR‐502‐3p was significantly elevated in the PSCI group compared to the PSCN group, and the subsequent ROC curve confirmed an area under ROC curve of 0.850. Existing studies have confirmed that miR‐502‐3p is often abnormally elevated in various diseases linked to cognitive impairment. For example, GABAergic neurons are the major inhibitory neurons in the brain, and their weakened function is associated with multiple neurodegenerative diseases (F. Yuan et al. 2015). Upregulated miR‐502‐3p can target the GABA A receptor subunit α‐1 (GABRα1) gene, inhibiting its expression and thereby affecting the cognitive function of Alzheimer's disease patients (Kumar et al. 2022). A recent study reports that miR‐502‐3p is primarily expressed in neurons and astrocytes, indicating these cell types as potential sources of the respective miRNAs (Devara et al. 2025). Moreover, miR‐502‐3p is related to oxidative stress and immune response. Specifically, accumulating research has demonstrated that the overexpression of miR‐502‐3p modulates the expression of genes associated with not only oxidative stress and immune response but also synaptic function (Sharma, Rodarte, Goyal, Rodriguez, et al. 2025). The abnormal expression of these genes may lead to neuroinflammation and neuronal damage, further exacerbating cognitive impairment. Additionally, the ROC curve plotted based on miR‐502‐3p confirmed its high sensitivity and specificity for diagnosing PSCI as a potential biomarker and provided a new avenue for the early identification and intervention of PSCI patients.

Subsequent work focused on the association of miR‐502‐3p and PSCI. Our assessment indicated that miR‐502‐3p level was positively correlated with both NIHSS and mRS scores, while it was negatively correlated with the MoCA score. The NIHSS measures stroke‐related neurological deficits, with higher scores indicating more severe impairments (Alawneh et al. 2022). Elevated miR‐502‐3p levels correlated positively with NIHSS scores, suggesting a link to more severe deficits. The mRS evaluates post‐stroke neurological recovery, with higher scores denoting greater disability. A positive correlation between elevated miR‐502‐3p and mRS scores implied that elevated miR‐502‐3p may be associated with poor overall neurological recovery in PSCI patients. In addition, the MoCA is a crucial tool for evaluating cognitive function, with lower scores reflecting more severe cognitive impairments (Gallucci et al. 2024). The negative correlation between miR‐502‐3p and MoCA scores suggested that increased miR‐502‐3p levels might be tied to more severe cognitive dysfunction. The above findings showed that miR‐502‐3p levels strongly correlated with both neurological deficits and cognitive impairments in PSCI patients, making it a potential biomarker for assessing PSCI severity.

Our multivariate logistic regression analysis manifested that the miR‐502‐3p and hypertension were independent risk factors for PSCI. Supportive evidence can be observed in published reports. Prior research has shown that hypertension is a major risk factor for stroke (Z. H. Lu et al. 2019; Y. Wang et al. 2021) and is closely linked to the PSCI (Y. Wang et al. 2021). In a previous report, hypertension has been confirmed to be closely related to the PSCI risk with an HR of 1.53 (H. Huang et al. 2024). It has also been indicated that hypertension worsens secondary neurodegeneration following stroke (Sayed et al. 2020). The impact of hypertension on PSCI may involve multiple aspects. It can cause structural and functional changes in cerebral blood vessels, like small artery sclerosis and endothelial damage, which impact blood flow and oxygen delivery to the brain, harming cognitive function. Long‐term hypertension may also lead to white matter lesions and brain atrophy, worsening cognitive impairments (Alfaro et al. 2018; Mok et al. 2024). In our study, miR‐502‐3p was also an important risk indicator for PSCI. Although no direct evidence indicates that miR‐502‐3p is associated with PSCI risk, reports on cognitive impairment‐related diseases offer referential evidence. For instance, miR‐502‐3p has been identified as a biomarker of vascular dementia due to cerebrovascular disease (Prabhakar et al. 2017). The impact of miR‐502‐3p on PSCI is likely mediated through the regulation of synaptic protein expression and synaptic function (Rivera et al. 2023; Sharma, Rodarte, Goyal, Rodriguez, et al. 2025). Additionally, research has indicated that aberrant miR‐502‐3p expression notably influences synaptic activity and dendritic spine density in the murine brain (Sharma, Rodarte, Goyal, Miranda, et al. 2025). Current reports provide strong evidence for the association of miR‐502‐3p and PSCI risk. However, our study was conducted based on a single‐center cohort, which may limit the general applicability of our research results. The patient sources, diagnostic procedures, and the way inclusion/exclusion criteria were implemented in the single center were highly consistent. This “consistency” would unintentionally screen out patients with specific characteristics, resulting in selection bias. This might overestimate the universal predictive value of miR‐502‐3p in PSCI, or miss the associations in key subgroups. In future, the present conclusion needs to be verified in a larger sample size in subsequent studies.

In summary, we found that significantly elevated serum miR‐502‐3p was a promising biomarker for the prognosis of PSCI. Elevated miR‐502‐3p and hypertension were independent risk factors for PSCI. Some limits can also be found in this study. The small sample size from a single hospital may limit the generalizability of the results. Unidentified confounding factors, like diet and lifestyle, could also affect the results. Additionally, the specific mechanisms of miR‐502‐3p in the pathogenesis of PSCI have not been explored. In future studies, we will consider expanding the sample size and collecting more potential confounding factors. And further investigation on the function of miR‐502‐3p in nerve cells and its impact on cognitive function is also necessary for revealing its mechanisms in PSCI.

## Author Contributions

**Yi Lin**: software, investigation, funding acquisition, writing – original draft, writing – review and editing. **Liang Xu**: data curation, investigation, validation, visualization, project administration, writing – review and editing. **Yuhao Zhang**: conceptualization, data curation, writing – original draft, writing – review and editing. **Cui Zou**: conceptualization, data curation, validation, formal analysis, visualization, project administration, writing – review and editing.

## Funding

This study received funding from Medical Research Project of Yancheng Health Commission (YK2025099).

## Conflicts of Interest

The authors declare no conflicts of interest.

## Data Availability

The datasets used and/or analyzed during the current study are available from the corresponding author on reasonable request.
